# Factors for the Primary Prevention of Breast Cancer: A Meta-Analysis of Prospective Cohort Studies

**DOI:** 10.34172/jrhs.2021.57

**Published:** 2021-07-20

**Authors:** Jalal Poorolajal, Fatemeh Heidarimoghis, Manoochehr Karami, Zahra Cheraghi, Fatemeh Gohari-Ensaf, Fatemeh Shahbazi, Bushra Zareie, Pegah Ameri, Fatemeh Sahraei

**Affiliations:** ^1^Department of Epidemiology, School of Public Health, Hamadan University of Medical Sciences, Hamadan, Iran; ^2^Modeling of Noncommunicable Diseases Research Center, Hamadan University of Medical Sciences, Hamadan, Iran; ^3^Research Center for Health Sciences, Hamadan University of Medical Sciences, Hamadan, Iran; ^4^Social Determinants of Health Research Center, Hamadan University of Medical Sciences, Hamadan, Iran

**Keywords:** Breast neoplasms, Risk factors, Behavior, Nutrition, Meta-analysis

## Abstract

**Background:** This report provided the effect of 15 preventable factors on the risk of breast cancer incidence.

**Study design:** A systematic review and meta-analysis.

**Methods:** A detailed research was conducted on PubMed, Web of Science, and Scopus databases in January 2020. Reference lists were also screened. Prospective cohort studies addressing the associations between breast cancer and 15 factors were analyzed. Between-study heterogeneity was investigated using the χ^2^, τ^2^, and I^2^ statistics. The probability of publication bias was explored using the Begg and Egger tests and trim-and-fill analysis. Effect sizes were expressed as risk ratios (RRs) with 95% confidence intervals (CIs) using a random-effects model.

**Results:** Based on the results, out of 147,083 identified studies, 197 were eligible, including 19,413,702 participants. The RRs (95% CI) of factors associated with breast cancer were as follows: cigarette smoking 1.07 (1.05, 1.09); alcohol drinking 1.10 (1.07, 1.12); sufficient physical activity 0.90 (0.86, 0.95); overweight/obesity in premenopausal 0.92 (0.82, 1.03) and postmenopausal 1.18 (1.13, 1.24); nulliparity 1.16 (1.03, 1.31); late pregnancy 1.37 (1.25, 1.50); breastfeeding 0.87 (0.81, 0.93); ever using oral contraceptive 1.00 (0.96, 1.05); ever using estrogen 1.13 (1.04, 1.23); ever using progesterone 1.02 (0.84, 1.24); ever using estrogen/progesterone 1.60 (1.42, 1.80); ever taking hormone replacement therapy 1.26 (1.20, 1.32); red meat consumption 1.05 (1.00, 1.11); fruit/vegetable consumption 0.87 (0.83, 0.90); and history of radiation therapy, based on single study 1.31 (0.87, 1.98).

**Conclusions:** This meta-analysis provided a clear picture of several factors associated with the development of breast cancer. Moreover, the useful information in this study may be utilized for ranking and prioritizing preventable risk factors to implement effective prevention programs.

## Introduction

Breast cancer is the most commonly occurring cancer in women, regardless of race or ethnicity, impacting over 2 million women worldwide annually, responsible for over 600,000 deaths in 2018 ^1^. According to the biennial report of the American Cancer Society, the breast cancer incidence rate had increased slightly by 0.3% per year ^2^. Several factors, such as genetic characteristics, lifestyle factors, and medical conditions, may play a role in the development of breast cancer. The risk factors for breast cancer can be divided into two categories, namely (a) fixed risk factors that may contribute to the development of breast cancer, however, cannot be changed, such as age, female gender, genetic characteristics ^3^, immunologic biomarkers ^4^, racial or ethnic characteristics ^5^, family history ^6^, and late menopause ^7^; and (b) modifiable risk factors that play a role in the development of breast cancer and can be changed, such as alcoholic beverage consumption^8^, cigarette smoking^9^, physical inactivity^10^, high body mass index (BMI) ^11^, high dietary fat^12^, and dietary fiber intake ^13^. These factors are largely modifiable and preventable, and therefore, can be considered when designing effective prevention programs.

 Efforts to improve screening programs and the early detection and treatment of breast cancer are important; nevertheless, it is of priority to take action to address preventable factors that play an important role in the development of breast cancer. Some measures, such as ranking and prioritizing the risk factors that contribute to the development of breast cancer and implementing prevention programs, can reduce the incidence of breast cancer and prevent thousands of new cases each year. Effective intervention strategies and prevention programs need a comprehensive understanding and a clear picture of the contributing factors that promote breast cancer. To the best of our knowledge, no comprehensive systematic review has yet been conducted to address all the potential preventable factors playing a pivotal role in the development of breast cancer. This meta-analysis was performed to address the associations between breast cancer and 15 factors that might be potentially modifiable and preventable, and consequently, might provide an opportunity to be addressed in prevention programs aimed to reduce the incidence of breast cancer.

## Methods

 The eligibility criteria in this study were based on population, intervention, comparison, outcomes, and study design (PICOS). Accordingly, the women having any of the 15 preventable factors mentioned below were included in the exposure group and those without any of the 15 preventable factors mentioned below in the unexposed group. The outcome was considered breast cancer and the prospective cohort studies were reviewed.

###  Eligibility criteria

 The outcome of interest was having pathologically confirmed breast cancer, of any type (i.e., ductal or lobular carcinomas), among the general population, regardless of age, gender, race, ethnicity, and geographical region. The exposures of interest are listed below:

- Cigarette smoking (current/former smokers versus nonsmokers)
- Drinking alcohol (current/former drinkers versus non-drinkers)
- Physical activity (sufficient versus insufficient)
- Body mass index (overweight/obese versus normal weight)
- Parity (nulliparous versus primiparous/multiparous)
- Late pregnancy (≥30 years versus <30 years)
- Breastfeeding (ever versus never, or ≥6 months versus <6 months, or ≥12 months versus <12 months, or ≥24 months versus <24 months)
- Ever using oral contraceptive (OCP) (yes versus no)
- Ever using estrogen (yes versus no)
- Ever using progesterone (yes versus no)
- Ever using estrogen/progesterone (yes versus no)
- Ever taking hormone replacement therapy (HRT) (yes versus no)
- Intake of red meat (highest intake versus lowest intake)
- Intake of fruit/vegetable (highest intake versus lowest intake)
- History of radiation therapy (yes versus no)

A BMI of 18.5-24.9 kg/m^2^ was classified as normal weight, 25.0-29.9 kg/m^2^ as overweight, and ≥ 30.0 kg/m^2^ as obese. At least 30 minutes of moderate- to vigorous-intensity physical activity per day (or 150 minutes per week) was considered sufficient for adults ^14^. Pregnancy over the age of 30 is considered high-risk ^15^. Accordingly, reproductive ages of > 30 were considered late pregnancy in this study. The duration of breastfeeding is recommended for at least 6 months continued up to 2 years of age or longer ^16^. Accordingly, various periods of breastfeeding, including ≥ 6, ≥ 12, and ≥ 24 months were considered in this research. The consumption of at least five total servings (400 grams) of fruit and vegetables per day is recommended ^17^. However, the majority of the included studies did not report fruit and vegetable consumption according to the recommendations of the World Health Organization. Therefore, the highest intake versus the lowest intake of fruit and vegetables were compared in the present study. There is no universal recommendation for red meat consumption. In this respect, the highest intake versus the lowest intake of red meat was also compared in this research.

 Prospective cohort studies addressing the association between breast cancer and any of the above factors were included in the meta-analysis, irrespective of their language and publication date and the participants' nationality, race, gender, and age. Wherever reported, full adjusted forms of risk ratio (RR) controlled was used for at least one or more potential confounding factors.

###  Information sources and search

 A detailed search was conducted on PubMed, Web of Science, and Scopus databases in January 2020. The reference lists of the included studies were also explored. The search process was performed based on the following keywords: (Breast cancer or Breast neoplasms or Breast malignancy or Breast tumor) and (Smoking or Cigarette or Tobacco or Cigar or Alcohol or Ethanol or Exercise or Physical activity or Obese or Obesity or Overweight or Body mass index or BMI or Pregnancy or Breastfeeding or Contraceptive or Hormone or Estrogen or Progesterone or Fruit or Vegetables or Red meat or Radiation)

###  Study selection

 The search results of all databases were combined using EndNote, and duplicates were deleted. Afterward, six authors (i.e., FH, FS, BZ, PA, FS, and FG) formed three two-person groups. Each group screened the titles and abstracts of one-third of the search results separately and independently and excluded ineligible studies. The full texts of potentially relevant studies were retrieved for further evaluation.

###  Data extraction

 The data from the relevant studies were extracted by 6 authors (i.e., FH, FS, BZ, PA, FS, and FG) using an electronic data collection form prepared in Stata (StataCorp, College Station, TX, USA).

###  Methodological quality

The Newcastle-Ottawa Scale (NOS) ^18^ was used to assess the methodological quality of the included studies. Based on this scale, a maximum of 9 stars were assigned to each study. Studies that received 7 or more stars were labeled high-quality; otherwise, studies were classified as low-quality.

###  Heterogeneity and publication bias

The heterogeneity across studies was examined using the Chi-square (χ^2^) test ^19^ and tau-square (τ^2^) test and quantified by the I^2^ statistic ^20^. According to the I^2^ value, heterogeneity was classified as low (<50%), moderate (50-74%), or high (≥75%). The possibility of publication bias was explored by the Egger ^21^ and Begg ^22^ tests and the trim-and-fill method ^23^.

###  Summary measures

The effect measure of choice was the RR with 95% confidence intervals. The results were reported based on a random-effects model^24^. The data were analyzed at a significance level of 0.05 using Stata software (version 14.2; StataCorp, College Station, TX, USA) and Review Manager software (version 5.3).

###  Sensitivity analysis

If the between-study heterogeneity was moderate to high (I^2^≥50%), the source of heterogeneity was investigated using the MetaPlot Stata command based on the sequential algorithm^25-27^.

## Results

###  Description of studies

**Figure 1:**
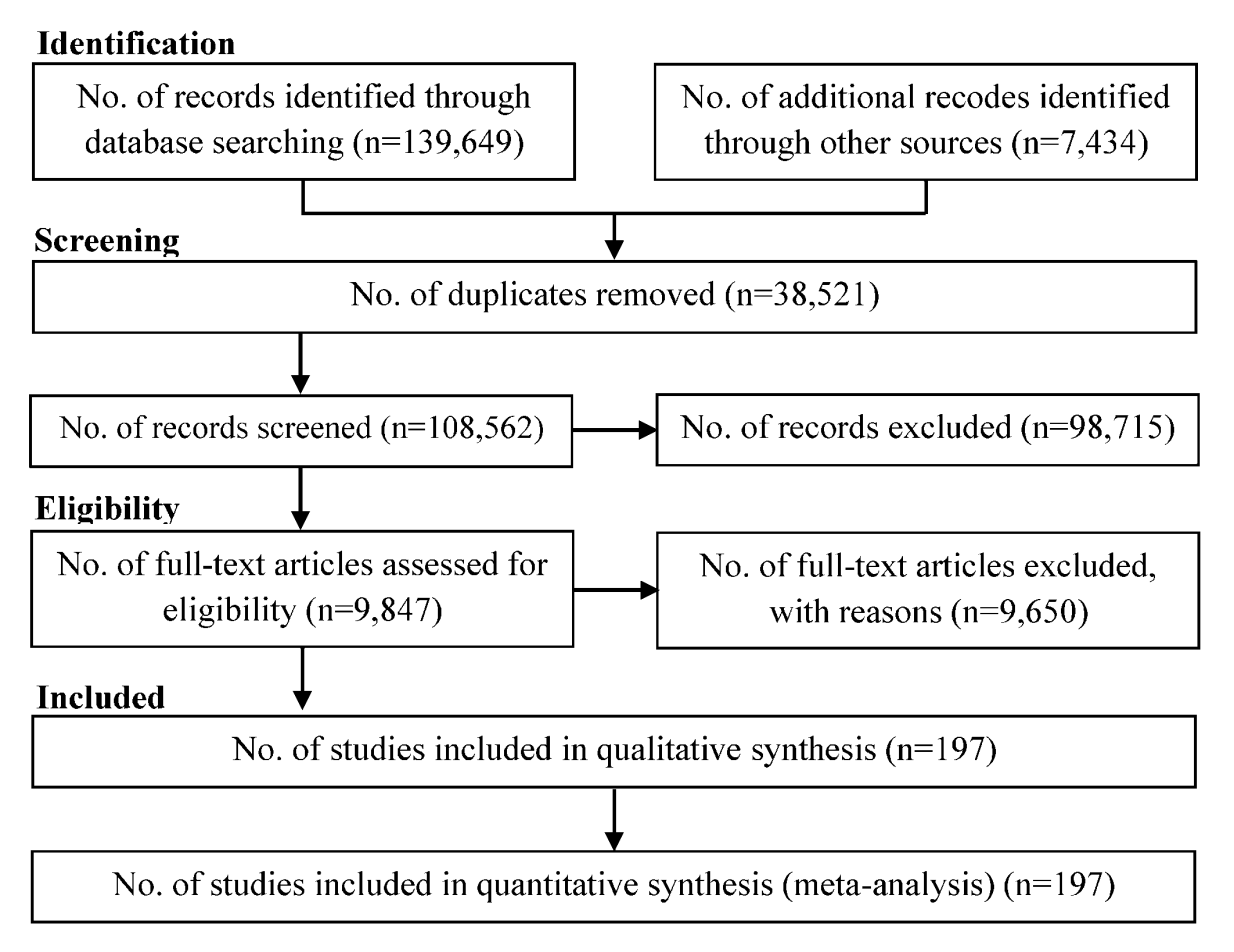
Flow of information through the various phases of the systematic review

In total, 147,083 studies were identified, including 139,649 studies obtained by searching the electronic databases in January 2020 and 7,434 articles identified by searching the reference lists of the included studies. After excluding duplicates and ineligible studies, 197 studies with 19,413,702 participants (Table 1) were included in the meta-analysis (Figure 1).

**Table 1:** Characteristics of the included studies (sorted by authors’ names)

**Row**	**1** ^St^ ** author, year**	**Country**	**Age (year)**	**Study design**	**Effect size**	**Adjustment**	**Sample Size**	**NOS Quality**
1	Adebamowo, 2005	USA	20-46	Prospective cohort	Rate Ratio	Adjusted	90,638	8- High quality
2	Agurs-Collins, 2009	USA	21-69	Prospective cohort	Rate Ratio	Adjusted	50,778	9- High quality
3	Al-Ajmi, 2018	UK	56.3	Prospective cohort	Risk Ratio	Adjusted	273,467	9- High quality
4	Albrektsen, 2005	Norway	20-74	Prospective cohort	Rate Ratio	Unadjusted	1,700,000	6- Low quality
5	Al-Delaimy, 2004	USA	25-42	Prospective cohort	Rate Ratio	Adjusted	116,671	8- High quality
6	Alipour, 2019	Iran	40-75	Nested case-control	Rate Ratio	Adjusted	499	8- High quality
7	Anderson, 2018	UK	40-69	Prospective cohort	Rate Ratio	Adjusted	262,195	9- High quality
8	Arslan, 2014	USA	60.05	Nested case-control	Rate Ratio	Adjusted	998	7- High quality
9	Arthur, 2017	USA	21-85	Nested case-control	Rate Ratio	Adjusted	1,052	8- High quality
10	Azam, 2018	Denmark	50-69	Prospective cohort	Hazard Ratio	Adjusted	4,501	8- High quality
11	Baglietto, 2010	Australia	27-81	Case-cohort	Risk Ratio	Unadjusted	1,054	9- High quality
12	Baglietto, 2011	Australia	40-69	Prospective cohort	Rate Ratio	Adjusted	20,967	8- High quality
13	Bakken, 2011	Europe	58.1	Prospective cohort	Risk Ratio	Adjusted	133,744	9- High quality
14	Barlow, 2006	USA	35-84	Prospective cohort	Risk Ratio	Adjusted	2,392,998	9- High quality
15	Bassett, 2013	Melbourne	27-80	Case-cohort	Risk Ratio	Adjusted	20,756	8- High quality
16	Bellocco, 2016	Sweden	56.1	Prospective cohort	Hazard Ratio	Adjusted	19,196	9- High quality
17	Beral, 2011	UK	56.6	Prospective cohort	Risk Ratio	Adjusted	1,129,025	8- High quality
18	Bergkvist, 1989	Sweden	≥35	Prospective cohort	Risk Ratio	Adjusted	23,244	6- Low quality
19	Bjerkaas, 2013	Norway	44	Prospective cohort	Hazard Ratio	Adjusted	302,865	8- High quality
20	Bjørge, 2010	Norway-Sweden-Austria	44	Prospective cohort	Risk Ratio	Adjusted	287,320	8- High quality
21	Bravi, 2018	Italy	41-79	Nested case-control	Rate Ratio	Adjusted	13,212	8- High quality
22	Brinton, 2013	Israel	31.1	Prospective cohort	Hazard Ratio	Adjusted	87,403	9- High quality
23	Brinton, 2014	USA	50-71	Prospective cohort	Hazard Ratio	Adjusted	190,827	8- High quality
24	Buring, 1987	USA	30-55	Prospective cohort	Risk Ratio	Adjusted	33,335	6- Low quality
25	Butler, 2010	Singapore	45-74	Prospective cohort	Rate Ratio	Adjusted	34,028	8- High quality
26	Butt, 2014	Sweden	57.23	Prospective cohort	Risk Ratio	Adjusted	14,092	9- High quality
27	Campa, 2011	USA-Europe	62.39	Nested case-control	Rate Ratio	Adjusted	20,468	7- High quality
28	Catsburg, 2015	Canada	40-59	Prospective cohort	Hazard Ratio	Adjusted	89,835	9- High quality
29	Cerhan, 1998	USA	65-102	Prospective cohort	Risk Ratio	Adjusted	1,806	7- High quality
30	Chen, 2002	USA	50-74	Nested case-control	Rate Ratio	Adjusted	1,397	7- High quality
31	Chen, 2016	Taiwan	≥35	Prospective cohort	Hazard Ratio	Adjusted	1,393,985	9- High quality
32	Chlebowski, 2013	USA	50-79	Prospective cohort	Hazard Ratio	Adjusted	41,449	8- High quality
33	Cho, 2006	USA	26-46	Prospective cohort	Risk Ratio	Adjusted	90,659	9- High quality
34	Clavel-Chapelon, 2007	France	40-65	Prospective cohort	Risk Ratio	Adjusted	80,377	8- High quality
35	Cohen, 2013	USA	40-79	Nested case-control	Rate Ratio	Adjusted	2,730	7- High quality
36	Colditz, 2003	USA	25-42	Prospective cohort	Risk Ratio	Adjusted	116,671	9- High quality
37	Cottet, 2009	France	52.2	Prospective cohort	Rate Ratio	Adjusted	65,374	9- High quality
38	Couto, 2013	Sweden	30-49	Prospective cohort	Risk Ratio	Adjusted	49,258	9- High quality
39	Cross, 2007	USA	50-71	Prospective cohort	Rate Ratio	Adjusted	500,000	9- High quality
40	Cust, 2009	Sweden	50-69	Nested case-control	Rate Ratio	Adjusted	1,122	7- High quality
41	Dai, 2009	China	40-70	Nested case-control	Rate Ratio	Adjusted	1,288	8- High quality
42	Dallal, 2007	USA	20-79	Prospective cohort	Risk Ratio	Adjusted	110,599	9- High quality
43	Dartois, 2016	France	42-72	Prospective cohort	Risk Ratio	Adjusted	67,634	8- High quality
44	Diallo, 2018	France	≥35	Prospective cohort	Rate Ratio	Adjusted	61,476	8- High quality
45	Diergaarde, 2008	USA	50-76	Nested case-control	Rate Ratio	Adjusted	975	6- Low quality
46	Dorgan, 1994	USA	35-68	Prospective cohort	Risk Ratio	Adjusted	2,321	8- High quality
47	Dorgan, 2010	Columbia	31-56	Nested case-control	Rate Ratio	Adjusted	266	9- High quality
48	Dossus, 2014	Europe	-	Prospective cohort	Hazard Ratio	Adjusted	322,988	9- High quality
49	Dumeaux, 2004	Norway	30-70	Prospective cohort	Risk Ratio	Adjusted	86,948	9- High quality
50	Dumeaux, 2005	France	40-64	Prospective cohort	Risk Ratio	Adjusted	68,670	7- High quality
51	Egan, 2002	USA	30-55	Prospective cohort	Risk Ratio	Adjusted	78,206	8- High quality
52	Egeberg, 2008	Denmark	50-64	Prospective cohort	Rate Ratio	Adjusted	24,697	8- High quality
53	Eisen, 2008	USA	58.2	Nested case-control	Rate Ratio	Adjusted	472	6- Low quality
54	Elebro, 2014	Sweden	-	Prospective cohort	Hazard Ratio	Adjusted	17,035	9- High quality
55	Ellingjord-Dale, 2017	Norway	50-69	Nested case-control	Rate Ratio	Adjusted	29,162	9- High quality
56	Epplein, 2009	USA	45-75	Nested case-control	Rate Ratio	Adjusted	821	7- High quality
57	Fabre, 2007	France	51.8	Prospective cohort	Risk Ratio	Adjusted	73,664	8- High quality
58	Fagherazzi, 2015	France	40-65	Prospective cohort	Hazard Ratio	Adjusted	66,481	9- High quality
59	Falk, 2014	USA	55-74	Prospective cohort	Hazard Ratio	Adjusted	54,562	9- High quality
60	Farhat, 2011	USA	50-79	Case-cohort	Risk Ratio	Adjusted	903	9- High quality
61	Farvid, 2014	USA	26-45	Prospective cohort	Rate Ratio	Adjusted	88,804	9- High quality
62	Feigelson, 2004	USA	50-74	Prospective cohort	Rate Ratio	Adjusted	97,786	8- High quality
63	Ferrucci, 2009	USA	55-74	Prospective cohort	Rate Ratio	Adjusted	52,158	9- High quality
64	Fourkala, 2014	UK	64	Prospective cohort	Hazard Ratio	Adjusted	92,834	7- High quality
65	Fournier, 2014b	France	59.68	Prospective cohort	Hazard Ratio	Adjusted	78,353	9- High quality
66	Fraser, 1997	USA	55	Prospective cohort	Risk Ratio	Adjusted	34,198	8- High quality
67	Friedenretch, 1993	Canada	No data	Nested case-control	Rate Ratio	Adjusted	1,701	8- High quality
68	Fuhrman, 2012	UK	55-74	Nested case-control	Rate Ratio	Adjusted	700	8- High quality
69	Fung, 2005	USA	30-55	Prospective cohort	Risk Ratio	Adjusted	11,700	8- High quality
70	Gapstur, 1999	USA	55-69	Prospective cohort	Risk Ratio	Adjusted	41,837	8- High quality
71	Garland, 1999	USA	25-42	Prospective cohort	Rate Ratio	Adjusted	116,671	8- High quality
72	Gaudet, 2014	USA	No data	Prospective cohort	Risk Ratio	Adjusted	28,965	9- High quality
73	Genkinger, 2013	USA	21-69	Prospective cohort	Rate Ratio	Adjusted	52,062	9- High quality
74	Gertig, 2006	Australia	40-69	Prospective cohort	Hazard Ratio	Adjusted	24,479	8- High quality
75	Goodman, 1997	Japan	No data	Prospective cohort	Risk Ratio	Adjusted	22,200	9- High quality
76	Gram, 2005	Norway-Sweden	30-50	Prospective cohort	Risk Ratio	Adjusted	102,098	9- High quality
77	Gram, 2015	USA	45-75	Prospective cohort	Hazard Ratio	Adjusted	83,300	9- High quality
78	Gram, 2016	Norway	34-70	Prospective cohort	Hazard Ratio	Adjusted	130,053	9- High quality
79	Ha, 2007	USA	22-92	Prospective cohort	Hazard Ratio	Adjusted	56,042	9- High quality
80	Hanaoka, 2005	Japan	40-59	Prospective cohort	Risk Ratio	Adjusted	27,398	9- High quality
81	Hankinson, 1997	USA	30-55	Prospective cohort	Risk Ratio	Adjusted	121,700	7- High quality
82	Hiatt, 1988	USA	No data	Prospective cohort	Risk Ratio	Adjusted	68,674	7- High quality
83	Holmberg, 1995	Sweden	40-75	Nested case-control	Rate Ratio	Adjusted	728	7- High quality
84	Holmes, 2003	USA	30-55	Prospective cohort	Risk Ratio	Adjusted	88,647	8- High quality
85	Horn, 2013	Norway	28-73	Prospective cohort	Hazard Ratio	Adjusted	58,426	9- High quality
86	Horn, 2014b	Norway	48-64	Prospective cohort	Hazard Ratio	Adjusted	21,532	8- High quality
87	Horn-Ross, 2004	USA	<85	Prospective cohort	Risk Ratio	Adjusted	103,460	9- High quality
88	Inoue-Choi, 2016	USA	24-43	Prospective cohort	Rate Ratio	Adjusted	193,742	9- High quality
89	Jick, 1980	USA	31-55	Prospective cohort	Risk Ratio	Adjusted	40,531	6- Low quality
90	Jones, 2017	UK	47	Prospective cohort	Hazard Ratio	Adjusted	102,927	8- High quality
91	Jordan, 2009	Thailand	28-51	Nested case-control	Rate Ratio	Adjusted	903	5- Low quality
92	Kabat, 2007	USA	40-59	Prospective cohort	Rate Ratio	Adjusted	49,654	9- High quality
93	Kabat, 2010	USA-UK-Canada	No data	Nested case-control	Rate Ratio	Adjusted	1,357	8- High quality
94	Kawai, 2010	Japan	40-64	Prospective cohort	Hazard Ratio	Adjusted	24,064	8- High quality
95	Kerlikowske, 2010	USA	56.4	Prospective cohort	Risk Ratio	Adjusted	587,369	8- High quality
96	Kim, 2012	USA	45-75	Nested case-control	Rate Ratio	Adjusted	1,426	7- High quality
97	Kim, 2017	Korea	≥30	Prospective cohort	Risk Ratio	Adjusted	5,046	9- High quality
98	Kojima, 2017	Japan	70-79	Prospective cohort	Rate Ratio	Adjusted	23,172	8- High quality
99	Komaroff, 2016	USA	≥50	Nested case-control	Rate Ratio	Adjusted	158	7- High quality
100	Kops, 2018	Brazil	40-69	Nested case-control	Rate Ratio	Adjusted	216	7- High quality
101	Kotsopoulos, 2010	USA	30-55	Prospective cohort	Rate Ratio	Adjusted	107,759	8- High quality
102	Krishnan, 2013	Australia	40-69	Prospective cohort	Hazard Ratio	Adjusted	14,441	8- High quality
103	Lahmann, 2007	Europe	20-80	Prospective cohort	Hazard Ratio	Adjusted	218,169	6- Low quality
104	Lambe, 1998	Sweden	<65	Nested case-control	Rate Ratio	Adjusted	8,205	6- Low quality
105	Lando, 1999	USA	55.5	Prospective cohort	Risk Ratio	Adjusted	5,761	8- High quality
106	Larsen, 2010	Denmark	50-64	Nested case-control	Rate Ratio	Adjusted	1,618	7- High quality
107	Larsson, 2009	Sweden	60.8	Prospective cohort	Rate Ratio	Adjusted	61,433	9- High quality
108	Lecarpentier, 2012	France	40.4	Prospective cohort	Risk Ratio	Adjusted	1,337	8- High quality
109	Lee, 2006	USA	45-75	Prospective cohort	Risk Ratio	Adjusted	55,371	8- High quality
110	Lee, 2014	Singapore	45-74	Nested case-control	Rate Ratio	Adjusted	1,623	8- High quality
111	Leon, 1989	UK	16-59	Prospective cohort	Rate Ratio	Adjusted	113,263	7- High quality
112	Lew, 2009	USA	50-71	Prospective cohort	Risk Ratio	Adjusted	184,418	8- High quality
113	Lin, 2008	Japan	40-79	Prospective cohort	Hazard Ratio	Adjusted	34,401	9- High quality
114	Link, 2013	USA	≤84	Prospective cohort	Rate Ratio	Adjusted	91,779	9- High quality
115	Lipnick, 1986	USA	30-55	Prospective cohort	Risk Ratio	Adjusted	121,964	6- Low quality
116	Liu, 2013	USA	25-44	Prospective cohort	Risk Ratio	Adjusted	91,005	9- High quality
117	Liu, 2016	Taiwan	45-64	Prospective cohort	Hazard Ratio	Adjusted	15,863	8- High quality
118	London, 1989	USA	30-55	Prospective cohort	Rate Ratio	Adjusted	117,557	7- High quality
119	Lowery, 2011	USA	>40	Prospective cohort	Risk Ratio	Adjusted	208,667	8- High quality
120	Lukanova, 2008	Sweden	30.96	Nested case-control	Rate Ratio	Adjusted	567	7- High quality
121	Luo, 2011	USA	50-79	Prospective cohort	Hazard Ratio	Adjusted	79,990	8- High quality
122	Ma, 2010	USA	No data	Prospective cohort	Risk Ratio	Adjusted	52,464	8- High quality
123	Manjer, 2000	Sweden	No data	Prospective cohort	Risk Ratio	Adjusted	10,902	8- High quality
124	Margolis, 2005	Norway-Sweden	30-49	Prospective cohort	Rate Ratio	Adjusted	99,504	8- High quality
125	Masala, 2017	Italy	35-64	Nested case-control	Rate Ratio	Adjusted	771	7- High quality
126	Mccarty, 2012	USA	55-74	Nested case-control	Rate Ratio	Adjusted	2,111	6- Low quality
127	Mertens, 2006	USA	45-64	Prospective cohort	Hazard Ratio	Adjusted	7,994	8- High quality
128	Michels, 1996	USA	30-55	Prospective cohort	Rate Ratio	Adjusted	121,701	9- High quality
129	Mills, 1989b	USA	55.4	Prospective cohort	Rate Ratio	Adjusted	20,341	7- High quality
130	Missmer, 2002	USA	31-90	Prospective cohort	Rate Ratio	Adjusted	351,041	9- High quality
131	Moradi, 2002	Sweden	25-50	Prospective cohort	Risk Ratio	Adjusted	25,778	7- High quality
132	Morimoto, 2002	USA	50-79	Prospective cohort	Risk Ratio	Adjusted	85,917	8- High quality
133	Nitta, 2016	Japan	40-79	Prospective cohort	Hazard Ratio	Adjusted	38,610	8- High quality
134	Nyante, 2014	USA	50-71	Prospective cohort	Hazard Ratio	Adjusted	186,150	9- High quality
135	Olsson, 2003	Sweden	25-65	Prospective cohort	Hazard Ratio	Adjusted	28,378	9- High quality
136	Opatrny, 2008	UK	50-75	Nested case-control	Rate Ratio	Adjusted	37,863	7- High quality
137	Ozmen, 2008	Turkey	18-70	Nested case-control	Rate Ratio	Adjusted	3,659	5- Low quality
138	Pala, 2009	Italy	25-70	Prospective cohort	Rate Ratio	Adjusted	319,826	9- High quality
139	Park, 2014	USA	45-75	Prospective cohort	Hazard Ratio	Adjusted	85,089	9- High quality
140	Persson, 1999	Sweden	No data	Prospective cohort	Risk Ratio	Adjusted	10,472	9- High quality
141	Phipps, 2012	USA	40-84	Prospective cohort	Hazard Ratio	Adjusted	1,054,466	8- High quality
142	Pijpe, 2010	Netherlands	44.5	Prospective cohort	Risk Ratio	Adjusted	725	8- High quality
143	Pijpe, 2012	France-UK-Netherlands	>18	Prospective cohort	Risk Ratio	Adjusted	1,993	6- Low quality
144	Poosari, 2014	Thailand	30-69	Prospective cohort	Hazard Ratio	Adjusted	11,414	9- High quality
145	Pouchieu, 2014	France	48.15	Prospective cohort	Rate Ratio	Adjusted	4,684	7- High quality
146	Reynolds, 2004	USA	No data	Prospective cohort	Hazard Ratio	Adjusted	116,544	9- High quality
147	Rice, 2016	USA	32-70	Nested case-control	Rate Ratio	Adjusted	4,712	7- High quality
148	Rintala, 2003	Finland	>25	Prospective cohort	Rate Ratio	Adjusted	10,049	8- High quality
149	Risch, 1994	Canada	43-49	Prospective cohort	Risk Ratio	Adjusted	33,003	7- High quality
150	Rockhill, 1999	USA	30-55	Prospective cohort	Risk Ratio	Adjusted	85,364	8- High quality
151	Rod, 2009	Denmark	62	Prospective cohort	Hazard Ratio	Adjusted	5,054	9- High quality
152	Rohan, 2000	Canada	40-59	Case-cohort	Risk Ratio	Adjusted	56,837	8- High quality
153	Romieu, 1989	USA	30-55	Prospective cohort	Risk Ratio	Adjusted	118,273	6- Low quality
154	Saxena, 2010	USA	60.82	Prospective cohort	Rate Ratio	Adjusted	56,867	8- High quality
155	Schairer, 1994	USA	57.4	Prospective cohort	Rate Ratio	Adjusted	49,017	7- High quality
156	Schatzkin, 1987	USA	25-74	Prospective cohort	Risk Ratio	Adjusted	7,188	9- High quality
157	Schoemaker, 2014	UK	No data	Nested case-control	Rate Ratio	Adjusted	608	7- High quality
158	Schuurman, 1995	Netherlands	55-69	Prospective cohort	Rate Ratio	Adjusted	62,573	7- High quality
159	Sellers, 1992	USA	55-69	Prospective cohort	Risk Ratio	Adjusted	37,105	7- High quality
160	Setiawan, 2009	USA	45-75	Prospective cohort	Risk Ratio	Adjusted	84,427	8- High quality
161	Shannon, 2005	China	50-64	Prospective cohort	Rate Ratio	Adjusted	1,070	8- High quality
162	Shin, 2016	Japan	50-70	Prospective cohort	Rate Ratio	Adjusted	49,552	9- High quality
163	Shore, 2008	USA	35-65	Nested case-control	Rate Ratio	Adjusted	1,224	7- High quality
164	Sieri, 2009	Italy	35-69	Nested case-control	Rate Ratio	Adjusted	837	8- High quality
165	Simon, 1991	USA	≥21	Prospective cohort	Risk Ratio	Adjusted	1,954	9- High quality
166	Sonestedt, 2008	Sweden	46-75	Prospective cohort	Risk Ratio	Adjusted	15,773	8- High quality
167	Sonnenschein, 1999	USA	35-65	Prospective cohort	Risk Ratio	Adjusted	8,157	8- High quality
168	Stahlberg, 2004	Denmark	>44	Prospective cohort	Risk Ratio	Adjusted	10,874	6- Low quality
169	Stahr, 2019	USA	18-100	Prospective cohort	Risk Ratio	Adjusted	21,931	9- High quality
170	Stuebe, 2009	USA	25-42	Prospective cohort	Hazard Ratio	Adjusted	60,075	8- High quality
171	Suzuki, 2006	Sweden	64.6	Prospective cohort	Rate Ratio	Adjusted	51,823	8- High quality
172	Taylor, 2007	UK	35-69	Prospective cohort	Rate Ratio	Adjusted	35,372	9- High quality
173	Tehard, 2006	France	45-70	Prospective cohort	Rate Ratio	Adjusted	69,116	8- High quality
174	Terry, 2001	Sweden	40-76	Prospective cohort	Rate Ratio	Adjusted	61,463	9- High quality
175	Thomas, 2001	Iceland	20-81	Nested case-control	Rate Ratio	Adjusted	10,422	7- High quality
176	Thorbjarnardottir, 2014	Iceland	59.2	Prospective cohort	Hazard Ratio	Adjusted	16,928	9- High quality
177	Thune, 1997	Norway	20-54	Prospective cohort	Risk Ratio	Adjusted	25,624	9- High quality
178	Tikk, 2015	Europe	54.8	Nested case-control	Rate Ratio	Adjusted	614	7- High quality
179	Tjønneland, 2004b	Denmark	50-64	Prospective cohort	Rate Ratio	Adjusted	23,618	7- High quality
180	Trapido, 1981	USA	25-57	Prospective cohort	Risk Ratio	Adjusted	95,519	7- High quality
181	Trieu, 2017	Vietnam	48.09	Nested case-control	Rate Ratio	Adjusted	788	6- Low quality
182	Tryggvadottir, 1997	Iceland	18-43	Nested case-control	Rate Ratio	Adjusted	1,387	8- High quality
183	Tulinius, 1990	Iceland	No data	Prospective cohort	Risk Ratio	Adjusted	61,040	5- Low quality
184	van den Brandt, 2017	Netherland	55-69	Case-cohort	Risk Ratio	Adjusted	62,573	8- High quality
185	van der Hel, 2004	Netherlands	20-59	Prospective cohort	Rate Ratio	Adjusted	493	7- High quality
186	Vatten, 1992	Norway	20-49	Prospective cohort	Risk Ratio	Adjusted	29,981	9- High quality
187	Velie, 2005	USA	40-91	Prospective cohort	Rate Ratio	Adjusted	40,559	8- High quality
188	Voorrips, 2002	Netherlands	55-69	Prospective cohort	Rate Ratio	Adjusted	62,573	8- High quality
189	Wada, 2015	Japan	54.15	Prospective cohort	Hazard Ratio	Adjusted	15,719	9- High quality
190	Wang, 2015a	China	35.55	Nested case-control	Rate Ratio	Adjusted	129	7- High quality
191	Wang, 2015b	USA	30-55	Prospective cohort	Hazard Ratio	Adjusted	106,037	8- High quality
192	Ward, 2008	UK	45-75	Nested case-control	Rate Ratio	Adjusted	1,189	8- High quality
193	Weiderpass, 2004	Norway	30-49	Prospective cohort	Risk Ratio	Adjusted	99,717	8- High quality
194	White, 2017b	USA	35-74	Prospective cohort	Hazard Ratio	Adjusted	50,884	8- High quality
195	Willett, 1987	USA	34-59	Prospective cohort	Risk Ratio	Adjusted	121,700	8- High quality
196	Zeleniuch-jacquotte, 2012	USA	34-65	Nested case-control	Rate Ratio	Adjusted	1,039	7- High quality
197	Zhang, 1999	USA	12-62	Prospective cohort	Rate Ratio	Adjusted	5,048	8- High quality

**NOS**: Newcastle Ottawa Scale, HRT: Hormone replacement therapy, OCP: Oral contraceptive pill, PA: Physical activity

###  Synthesis of results 

*Cigarette smoking* **—** Based on 90 studies (Supplementary File 1), the overall RR for smokers versus nonsmokers was 1.07 (95% CI, 1.05, 1.09). The overall effect measure showed that smoking significantly increased the risk of breast cancer by 7% (P *<*0.001). Between-study heterogeneity was moderate (I^2^=54%). The overall effect became a bit stronger (RR, 1.08; 95% CI, 1.06, 1.10; I^2^=42%) after performing a sensitivity analysis (Table 2).

Based on 48 studies, the overall RR for current smokers versus never smokers was 1.06 (95% CI, 1.03, 1.10). The overall effect measure showed that current smoking significantly increased the risk of breast cancer by 6% (*P<*0.001). Between-study heterogeneity was moderate (I^2^=65%). The overall effect became a bit stronger (RR, 1.09; 95% CI, 1.05, 1.13; I^2^=49%) after performing a sensitivity analysis.

Based on 42 studies, the overall RR for former smokers versus never smokers was 1.07 (95% CI, 1.05, 1.10). The overall effect measure showed that former smoking significantly increased the risk of breast cancer by 7% (*P*<0.001). Between-study heterogeneity was low (I^2^=29%). There was no evidence of publication bias (*P*=0.222 and *P*=0.965 based on the Begg and Egger tests, respectively)

*Drinking alcohol* **—** Based on 56 studies (Supplementary File 2), the overall RR for drinkers versus nondrinkers was 1.10 (95% CI, 1.07, 1.12). The overall effect measure showed that drinking significantly increased the risk of breast cancer by 10% (*P<*0.001). Between-study heterogeneity was moderate (I^2^=63%). The overall effect became slightly weaker (RR, 1.08; 95% CI, 1.06, 1.11; I^2^=49%) after performing a sensitivity analysis (Table 2).

Based on 46 studies, the overall RR for current drinkers versus never drinkers was 1.09 (95% CI, 1.06, 1.12). The overall effect measure showed that current drinking significantly increased the risk of breast cancer by 9% (*P<*0.001). Between-study heterogeneity was moderate (I^2^=66%). The overall effect became slightly weaker (RR, 1.08; 95% CI, 1.05, 1.10; I^2^=50%) after performing a sensitivity analysis.

Based on 10 studies the overall RR for former drinkers versus never drinkers was 1.22 (95% CI, 1.07, 1.39). The overall effect measure showed that former drinking significantly increased the risk of breast cancer by 22% (*P*<0.001). Between-study heterogeneity was low (I^2^=43%). There was no evidence of publication bias (*P*=0.997 and *P*=0.211 based on the Begg and Egger tests, respectively).

**Table 2:** Results of sensitivity analysis

**Variables**	**Before the sensitivity analysis**				**After the sensitivity analysis**			
	**Studies**	**χ** ^2^	**I** ^2^	**RR (95% CI)**	**Studies**	**χ** ^2^	**I** ^2^	**RR (95% CI)**
Cigarette smoking	90	0.001	54%	1.07 (1.05, 1.09)	84	0.002	42%	1.08 (1.06, 1.10)
Alcohol drinking	56	0.001	63%	1.10 (1.07, 1.12)	53	0.001	49%	1.08 (1.06, 1.11)
Sufficient physical activity	16	0.001	63%	0.90 (0.86, 0.95)	15	0.030	45%	0.89 (0.85, 0.94)
Overweight/obesity	52	0.001	76%	1.10 (1.05, 1.14)	45	0.001	45%	1.11 (1.08, 1.15)
Nulliparity	67	0.001	97%	1.16 (1.03, 1.31)	60	0.001	44%	1.22 (1.18, 1.27)
Late pregnancy	37	0.001	90%	1.37 (1.25, 1.50)	36	0.002	41%	1.29 (1.23, 1.35)
Breastfeeding	35	0.001	82%	0.87 (0.81, 0.93)	32	0.450	1%	0.93 (0.91, 0.96)
Ever using oral contraceptive	45	0.001	64%	1.00 (0.96, 1.05)	42	0.008	38%	1.04 (1.01, 1.08)
Ever using estrogen	23	0.001	88%	1.13 (1.04, 1.23)	18	0.010	46%	1.09 (1.03, 1.16)
Ever using progesterone	5	0.020	67%	1.02 (0.84, 1.24)	3	0.910	0%	1.01 (0.94, 1.10)
Ever using estrogen/progesterone	17	0.001	95%	1.60 (1.42, 1.80)	8	0.050	50%	1.47 (1.37, 1.59)
Ever taking hormone replacement therapy	62	0.001	88%	1.26 (1.20, 1.32)	42	0.001	50%	1.27 (1.23, 1.32)
Red meat consumption	22	0.003	52%	1.05 (1.00, 1.11)	21	0.010	47%	1.06 (1.01, 1.12)
Fruit/vegetable consumption	14	0.550	0%	0.87 (0.83, 0.90)	A sensitivity analysis was not necessary.			

*Sufficient physical activity —* Based on 16 studies (Supplementary File 3), the overall RR for sufficient versus insufficient physical activity was 0.90 (95% CI, 0.86, 0.95). The overall effect measure showed that physical activity reduced significantly the risk of breast cancer by 9% (*P*<0.001). Between-study heterogeneity was moderate (I^2^=63%). The overall effect became slightly stronger (RR, 0.89; 95% CI, 0.85, 0.94; I^2^=45%) after performing a sensitivity analysis. There was no evidence of publication bias (*P*=0.677 and P=0.136 based on the Begg and Egger tests, respectively).

*Body mass index —* Based on 52 studies (Supplementary File 4), the overall RR for overweight/obesity versus normal weight was 1.10 (95% CI, 1.05, 1.14). The overall effect measure showed that overweight/obesity significantly increased the risk of breast cancer by 10% (*P*<0.001). Between-study heterogeneity was high (I^2^=76%). The overall effect became slightly stronger (RR, 1.11; 95% CI, 1.08, 1.14; I^2^=49%) after performing a sensitivity analysis (Table 2). There was no evidence of publication bias (*P*=0.917 and *P*=0.105 based on the Begg and Egger tests, respectively).

The effect of body mass index on the incidence risk of breast cancer was evaluated in pre- and post-menopausal periods separately. Based on 15 studies (Supplementary File 5), the overall RR for overweight/obesity versus normal weight in the premenopausal period was 0.92 (95% CI, 0.82, 1.03). The overall effect measure showed that overweight/obesity had no significant effect on the risk of breast cancer (*P*=0.140). Between-study heterogeneity was low (I^2^=50%). On the other hand, based on 24 studies (Supplementary File 6), the overall RR for overweight/obesity versus normal weight during the postmenopausal period was 1.18 (95% CI, 1.13, 1.24). The overall effect measure showed that overweight/obesity significantly increased the risk of breast cancer by 18% (*P*<0.001).

*Parity —* Based on 67 studies (Supplementary File 7), the overall RR for nulliparous versus primiparous/multiparous was 1.16 (95% CI, 1.03, 1.31). The overall effect measure showed that nulliparity significantly increased the risk of breast cancer by 16% (*P*<0.001). Between-study heterogeneity was high (I^2^=97%). The overall effect became slightly stronger (RR, 1.22; 95% CI, 1.18, 1.27; I^2^=44%) after performing a sensitivity analysis (Table 2).

**Figure 2:**
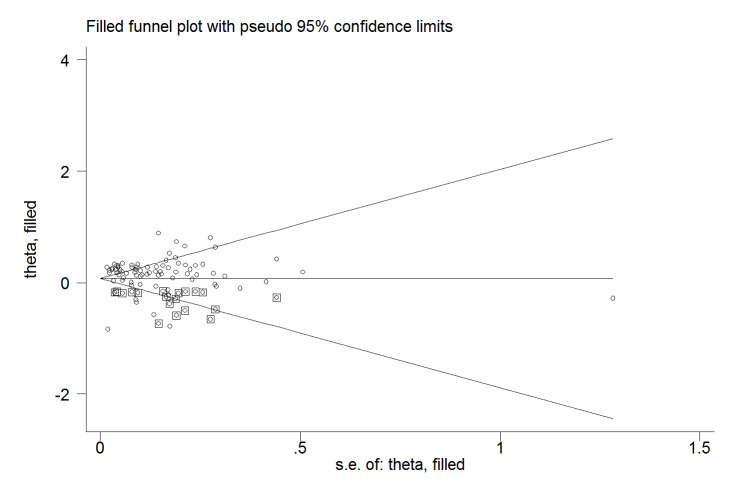
Trim-and-fill analysis estimating the number of possible missing studies for the association between breast cancer and nulliparity
 The squares represent the possible missing studies.

The Egger test revealed no evidence of publication bias (*P*=0.182); however, the Begg test did indicate evidence of publication bias (*P*=0.001). Trim-and-fill analysis estimated 19 missing studies (Figure 2) and the overall effect became slightly weaker (RR, 1.08; 95% CI, 0.99, 1.17).

*Late pregnancy —* Based on 37 studies (Supplementary File 8), the overall RR for late pregnancy of ≥30 years versus normal pregnancy of <30 years was 1.37 (95% CI, 1.25, 1.50). The overall effect measure showed that late pregnancy significantly increased the risk of breast cancer by 37% (*P<*0.001). Between-study heterogeneity was high (I^2^=90%). The overall effect became slightly weaker (RR, 1.29; 95% CI, 1.23, 1.35; I^2^=41%) after performing a sensitivity analysis (Table 2).

The Egger test revealed no evidence of publication bias (*P*=0.150); nevertheless, the Begg test did indicate evidence of publication bias (*P*=0.001); however, the trim-and-fill analysis estimated no missing studies.

*Breastfeeding —* Based on 35 studies Supplementary File 9, the overall RR for breastfeeding versus no breastfeeding was 0.87 (95% CI, 0.81, 0.93). The overall effect measure showed that breastfeeding reduced significantly the risk of breast cancer by 13% (*P*<0.001). Between-study heterogeneity was high (I^2^=82%). The overall effect became slightly weaker (RR, 0.93; 95% CI, 0.91, 0.96; I^2^=1%) after performing a sensitivity analysis (Table 2). There was no evidence of publication bias (*P*=0.178 and *P*=0.249 based on the Begg and Egger tests, respectively).

*Ever using OCP —* Based on 45 studies (Supplementary File 10), the overall RR for using OCP versus not using OCP was 1.00 (95% CI, 0.96, 1.05). Using OCP did not affect breast cancer (*P*=0.870). Between-study heterogeneity was moderate (I^2^=64%). The overall effect became slightly stronger and significant (RR, 1.04; 95% CI, 1.01, 1.08; I^2^=38%) after performing a sensitivity analysis (Table 2). There was no evidence of publication bias (*P*=0.417 and *P*=0.588 based on the Begg and Egger tests, respectively).

*Ever using estrogen —* Based on 23 studies (Supplementary File 11), the overall RR for using estrogen versus not using estrogen was 1.13 (95% CI, 1.04, 1.23). The overall effect measure showed that using estrogen significantly increased the risk of breast cancer by 13% (*P<*0.001). Between-study heterogeneity was high (I^2^=88%). The overall effect became slightly weaker (RR, 1.09; 95% CI, 1.03, 1.16; I^2^=46%) after performing a sensitivity analysis (Table 2). There was no evidence of publication bias (*P*=0.464 and *P*=0.913 based on the Begg and Egger tests, respectively).

*Ever using progesterone —* Based on 5 studies (Supplementary File 12), the overall RR for using progesterone versus not using progesterone was 1.02 (95% CI, 0.84, 1.24). The overall effect measure showed that using progesterone had no significant effect on breast cancer (*P=*0.820). Between-study heterogeneity was moderate (I^2^=67%). The overall effect became slightly weaker (RR, 1.01; 95% CI, 0.94, 1.10; I^2^=0%) after performing a sensitivity analysis (Table 2). There was no evidence of publication bias (*P*=0.293 and *P*=0.211 based on the Begg and Egger tests, respectively).

*Ever using estrogen/progesterone —* Based on 17 studies (Supplementary File 13), the overall RR for using estrogen/progesterone versus not using estrogen/progesterone was 1.60 (95% CI, 1.42, 1.80). The overall effect measure showed that using estrogen/progesterone significantly increased the risk of breast cancer by 60% (*P<*0.001). Between-study heterogeneity was high (I^2^=95%). The overall effect became slightly weaker (RR, 1.47; 95% CI, 1.37, 1.59; I^2^=50%) after performing a sensitivity analysis (Table 2). There was no evidence of publication bias (*P*=0.537 and *P*=0.528 based on the Begg and Egger tests, respectively).

*Ever taking hormone replacement therapy —* Based on 62 studies (Supplementary File 14), the overall RR for taking HRT versus not taking HRT was 1.26 (95% CI, 1.20, 1.32). The overall effect measure showed that taking HRT significantly increased the risk of breast cancer by 26% (*P<*0.001). Between-study heterogeneity was high (I^2^=88%). The overall effect became slightly stronger (RR, 1.27; 95% CI, 1.23, 1.32; I^2^=50%) after performing a sensitivity analysis (Table 2). There was no evidence of publication bias (*P*=0.775 and *P*=0.440 based on the Begg and Egger tests, respectively).

*Red meat consumption —* Based on 22 studies (Supplementary File 15), the overall RR for the highest intake versus the lowest intake of red meat was 1.05 (95% CI, 1.00, 1.11). The overall effect measure showed that the consumption of red meat had no significant effect on breast cancer (*P=*0.030). Between-study heterogeneity was moderate (I^2^=52%). The overall effect became slightly stronger (RR, 1.06; 95% CI, 1.01, 1.12; I^2^=47%) after performing a sensitivity analysis (Table 2). The Begg test revealed no evidence of publication bias (*P*=0.108), while the Egger test did indicate evidence of publication bias (*P*=0.022). However, the trim-and-fill analysis estimated no missing studies.

*Fruit/vegetable consumption —* Based on 14 studies (Supplementary File 16), the RR for the highest intake versus the lowest intake of fruit/vegetables infrequently was 0.87 (95% CI, 0.83, 0.90). The overall effect measure showed that fruit/vegetable consumption significantly reduced the risk of breast cancer by 23% (*P*=0.001). Between-study heterogeneity was low (I^2^=0%). There was no evidence of publication bias (*P*=0.412 and *P*=0.536 based on the Begg and Egger tests, respectively).

*History of radiation therapy —* Only one prospective cohort study^28^ was found that investigated the effect of previous radiation therapy on the incidence of breast cancer. Based on the results of this study, the RR for ever-exposing to radiation therapy versus never-exposing to radiation therapy was 1.31 (0.87, 1.98). The effect measure showed that exposure to radiation therapy had no significant effect on breast cancer.

###  Unified overview

**Figure 3:**
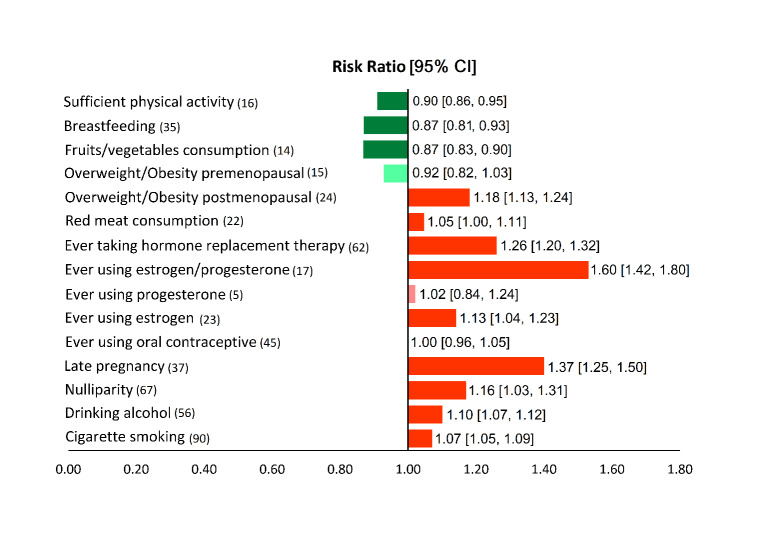
Associations (95% CIs) between breast cancer and nutritional and behavioral factors in a single view
 Protective factors are shown in green (dark green, significant) and risk factors are shown in red (dark red, significant; light red, non-significant). The figures in parenthesis show the number of included studies.

Figure 3 presents a unified overview of the associations between breast cancer and all nutritional and behavioral factors. As shown in this figure, taking HRT, using estrogen/progesterone, using estrogen, having late pregnancy, being nulliparous, consuming red meat, and being overweight/obese in the postmenopausal period were found to significantly increase the risk of breast cancer. In contrast, sufficient physical activity, fruit/vegetable consumption, and breastfeeding reduced significantly the risk of breast cancer. Meanwhile, exposure to ionizing radiation, using progesterone, using OCP, and being overweight/obese in the premenopausal period had no statistically significant effects on the risk of breast cancer.

## Discussion

According to our findings, estrogen/progesterone uptake and late pregnancy were the first and second most powerful risk factors for breast cancer, respectively, whereas, sufficient fruit/vegetable consumption and sufficient physical activity were the first and second most powerful protective factors against breast cancer, respectively. The magnitudes of the effect measures reported in this systematic review may be used for ranking and prioritizing the relative importance of risk and protective factors. However, it should be kept in mind that these factors vary in terms of their physiological modus operandi and their units of exposure; therefore, direct comparisons are often unwarranted ^29^. In other words, the mere fact that the RRs of some risk factors for breast cancer are higher than the RRs of other risk factors is not a sufficient basis for ranking and prioritizing risk factors. Instead, the prevalence of risk factors in the community is an essential criterion that needs to be taken into account when ranking and prioritizing risk factors. When the effect of a particular risk factor on the outcome of interest is strong (a high RR), however, the prevalence of that risk factor is low in the community, the overall impact of the risk factor on the disease burden in the community is low. In contrast, when a particular risk factor is common in the community, the overall impact of the factor on the outcome of interest may be tremendous even if the association between the risk factor and the outcome is not very strong (a low RR). Therefore, ranking and prioritizing the behavioral and nutritional factors affecting the risk of breast cancer depend on both the strength of the associations (the magnitude of RRs) and the prevalence of the factors in the community. Furthermore, it is impossible to consider risk or protective factors as separate elements, rather, they should be considered a collection. Risk factors facilitate the occurrence of diseases, while protective factors inhibit their occurrence. When a balance exists between risk and protective factors or when protective factors overcome risk factors, the disease will not occur. In contrast, the disease will occur when risk factors are stronger than protective factors ^30^.

Our results revealed a positive association between cigarette smoking and the development of breast cancer. Ambrosone et al. conducted a meta-analysis of observational studies in 2008 and reported the effect of cigarette packs/years on breast cancer risk. They reported a dose-dependent fashion RR=1.44 (95% CI: 1.23, 1.68 for ≥20 pack/years versus never smokers) ^31^. Cigarette smoke contains over 7,000 toxic chemical compounds, including human carcinogens ^32^. These toxins and carcinogens can result in direct DNA damage. Since DNA controls the normal growth and function of cells, damage to DNA can alter the growth patterns of cells. These abnormal gastric epithelial cells with DNA damage can turn into cancer^33,34^.

This meta-analysis indicated that drinking alcohol increased the risk of developing breast cancer. Acetaldehyde, the first and most toxic metabolite of ethanol, is a human carcinogen and can induce DNA lesions by inhibiting DNA methylation and by interacting with retinoid metabolism ^35^. DNA lesions can cause cell mutations, which can convert a normal cell into cancer ^36^. Moreover, alcohol can act as an irritant and lead to mucosal damage. The damaged cells may try to repair themselves, which may cause DNA changes that can be a step toward cancer ^37^.

According to our results, the risk of breast cancer of former drinkers was higher than that of current drinkers. One possible explanation for this finding is that former drinkers might be heavy drinkers who had drunk alcohol for many years; however, they were forced to quit drinking alcohol because of severe hepatobiliary and gastrointestinal complications ^38^. Consistent with our findings, Key et al. performed a meta-analysis of studies addressing the association between alcohol and breast cancer in 2006. They concluded that excess risk associated with alcohol drinking was 22% (95% CI: 9%, 37%); each additional 10 g ethanol/day was associated with a risk increase by 10% (95% CI: 5%, 15%) ^39^. In addition, Bagnardi et al. ^40^ conducted a dose-response meta-analysis to address the effect of alcohol consumption and site-specific cancer risk. Based on the results of the mentioned meta-analysis, the relative risk of female breast cancer was reported to be 1.04 (95%: 1.01, 1.07), 1.23 (95% CI: 1.19, 1.28), and 1.61 (95% CI: 1.33, 1.94) for light, moderate, and heavy drinking, respectively. Although the approach of this meta-analysis to address the effect of drinking alcohol on breast cancer risk was different from ours, its results were consistent with ours confirming that drinking alcohol can increase the risk of breast cancer.

Our results showed a positive and significant causal relationship between breast cancer and overweight and obesity as a whole. However, the effect of BMI on the incidence risk of breast cancer was different in pre- and post-menopausal periods separately. It was revealed that overweight/obesity had no significant effect on the risk of breast cancer in the premenopausal period (*P*<0.170); nonetheless, it had a significant impact on the postmenopausal period (*P*<0.001). These findings were consistent with the results of a previous meta-analysis conducted by Cheraghi et al. ^11^ in 2012. They reported that overweight and obesity had no significant effect on the incidence of breast cancer during the premenopausal period, whereas it might increase the postmenopausal risk of breast cancer. Evidence, based on the meta-analyses of observational studies, indicated that excess BMI not only increased the postmenopausal risk of breast cancer but also heightened the risk of gynecologic cancer in women, such as endometrial cancer ^41^, cervical cancer ^42^, and ovarian cancer ^43^.

Based on our findings, using OCP, estrogen, progesterone, a combination of estrogen/progesterone, and HRT significantly increased the risk of breast cancer. The results of several previously conducted meta-analyses approved our findings. Steinberg et al. conducted a meta-analysis of case-control studies using community controls that analyzed the effect of conjugated equine estrogens on breast cancer. They reported that the risk of breast cancer after 10 years of estrogen use increased by at least 15% and up to 29% ^44^. Based on the findings of another meta-analysis conducted by Steinberg et al., hormone replacement therapy using estradiol (with or without progestin) was associated with an increased risk of breast cancer RR=2.2 (95% CI, 1.4, 3.4) after 15 years ^45^. Several mechanisms have been suggested to explain the association by which HRT increases the risk of breast cancer. The results of experimental studies showed that rigorous cell proliferation occurs upon hormonal exposure in patients with hormone receptor-positive breast cancer. Zghair et al. indicated that breast cancer type 1 susceptibility protein (*BRCA1*) was the predominant marker gene responsible for estrogen regulation. They reported that exposure to high levels of estrogen, as well as exposure to high levels of iron during the postmenstrual period, exerted synergistic effects on cellular proliferation in *BRCA1*-linked hormone-responsive breast cancer ^46^. Additionally, both in vivo and in vitro investigations have been demonstrated that combination therapy with estradiol and estrogen/norethisterone increases the overexpression of proliferation of progesterone receptor membrane component 1 in breast cancer cells ^47^. Furthermore, Wiebe et al. reported that progesterone metabolite 5α-pregnane stimulated breast cell proliferation and detachment, and therefore, played an important role in the development of breast cancer ^48^.

The results of the present study indicated that breastfeeding decreased the risk of breast cancer by 13%, while late pregnancy significantly increased the risk of breast cancer by 40%. Consistent with our findings, Unar-Munguía recently conducted a meta-analysis in 2017 and showed that the relative risk for breast cancer in women who had breastfed exclusively was 0.72 (95% CI: 0.58, 0.90), compared to women who had never breastfed ^49^. On the other hand, Namiranian et al. showed in a meta-analysis that the age of first pregnancy after 30 years was associated with an increased risk of breast cancer odds ratio=1.52 (95% CI: 1.30, 1.77) ^50^. Pregnancy is associated with extensive changes to the breasts, making breast cells less likely to multiply and develop tumors. This issue explains the protective effect of pregnancy on younger women. However, after the age of 35 years, breast tissue is more likely to have accumulated cells carrying cancer-causing mutations, or clusters of abnormal cells with the potential to become cancerous. However, the important question is why the first pregnancy after age 35 increases the risk of breast cancer. The answer to this question lies in a signaling pathway called the JAK-STAT5 pathway. During pregnancy, pre-existing precancerous cells activate the PRLR-Jak2-STAT5 signaling pathway, accelerating their progression to fully cancerous cells. Blocking Jak2-STAT5 activity can reduce breast cancer risk associated with late-age pregnancy. This pathway can be blocked by various molecules, including Ruxolitinib, AG490, and C188-9 ^51^.

Our results indicated that sufficient physical activity significantly reduced the risk of breast cancer. Based on the findings of a meta-analysis conducted by Chen et al., there was an inverse association between physical activity and risk of breast cancer OR=0.87 (95% CI: 0.84, 0.90) ^52^. Another meta-analysis conducted by Wu et al. reported a dose-response inversed relationship between physical activity and breast cancer risk. According to the results of this meta-analysis, the risk of breast cancer decreased by 2% for every 25 metabolic equivalents (MET)-h/week increment in non-occupational physical activity, 3% for every 10 MET-h/week increments in a recreational activity, and 5% for every 2 h/week increments in moderate plus vigorous recreational activity ^53^. The mechanism by which physical activity reduces the risk of breast cancer is controversial. The results of empirical studies proposed that exercise-induced transient systemic acidosis will alter the in situ tumor microenvironment and delay tumor adaptation to regional hypoxia and acidosis in the later stages of carcinogenesis. Smallbone et al. demonstrated that repeated episodes of transient systemic acidosis would interrupt critical evolutionary steps in the later stages of carcinogenesis resulting in a substantial delay in the evolution of the invasive phenotype. They suggested that transient systemic acidosis might mediate the observed reduction in cancer risk associated with increased physical activity ^54^.

Based on our findings, the intake of fruit and vegetable had a significant protective effect against breast cancer. The results of a meta-analysis recently conducted by Zhang et al. showed that the intake of vegetable-fruit-soybean dietary patterns could reduce the risk of breast cancer RR=0.87 (95% CI: 0.82, 0.91) ^55^. Another meta-analysis conducted by Gandini et al. reported similar results. Based on the results of the mentioned meta-analysis, the relative risk of breast cancer for those who consumed vegetables was 0.75 (95% CI: 0.66, 0.85), and for those who consumed fruit was 0.94 (95% CI: 0.79, 1.11) ^56^. It has been postulated that the anti-carcinogenic effects of fruits and vegetables may be attributed to the antioxidant effect of their vitamin content, especially vitamin C and beta-carotene. Antioxidants neutralize reactive oxygen free radicals, which cause DNA damage ^57,58^, which in turn may result in genetic modifications and carcinogenesis ^,34^.

Based on our findings, red meat consumption had a weak, yet, significant positive association with breast cancer. Farvid et al. ^59^ conducted a meta-analysis to address the effect of red and processed meat consumption on breast cancer incidence. They concluded that red meat consumption was associated with a 6% higher breast cancer risk (RR=1.06; 95% CI: 0.99, 1.14). The findings of another meta-analysis conducted by Guo et al. showed similar results. They reported that the relative risk of breast cancer for the highest versus the lowest consumption of red meat was 1.10 (95% CI: 1.02, 1.19) ^60^. Current evidence recommends consuming no more than moderate amounts of red meat, such as beef, pork, and lamb, and eat little, if any, processed meat. The recommendation is to limit consumption to no more than about three portions per week, which are equivalent to about 350-500 grams (about 12-18 ounces) cooked weight of red meat ^61^. There is strong evidence that the intake of either red or processed meat is the cause of colorectal, stomach, and breast cancers ^38,62^.

 This review had a few limitations and potential biases. There were some studies, mostly old, that seemed potentially eligible to be included in this meta-analysis; nevertheless, neither their full texts nor their corresponding authors were accessible. This issue might have introduced a selection bias in our results. Furthermore, some epidemiological studies that addressed the associations between breast cancer and some risk factors were excluded from the meta-analysis since they were not consistent with the inclusion criteria defined for this review. This issue might also have raised the possibility of selection bias.

 Despite its limitations, this meta-analysis had three priorities over the previously conducted ones. First, many of the previous meta-analyses were carried out several years ago and needed to be updated based on current evidence. Second, in this study, 15 modifiable risk factors were examined, for some of which, no meta-analysis has been conducted before. Third, only the results of prospective cohort studies were employed that were the gold standard for observational studies with higher credibility.

## Conclusion

 This meta-analysis provided a clear picture of several factors playing pivotal roles in the development of breast cancer. These results are helpful and may be utilized for ranking and prioritizing preventable risk factors to implement effective interventions and community-based prevention programs. It is reemphasized that both the strength of associations and the prevalence of factors in the community should be taken into account when ranking and prioritizing breast cancer-associated factors.

## Acknowledgments

 These results were obtained as a part of an MSc thesis in Epidemiology. The authors would like to appreciate the Vice-Chancellor for Research and Technology of the Hamadan University of Medical Sciences, Hamadan, Iran, for approval and financial support of this study.

## Conflict of interests

 The authors have no conflict of interest to declare.

## Funding

 The Vice-Chancellor of Research and Technology, Hamadan University of Medical Sciences, funded this study (No. 9611247562). The funder had a role in the study design, data collection, analysis, publication decision, or manuscript preparation.

## Highlights

- Using estrogen/progesterone and late pregnancy are the first and second most powerful risk factors for breast cancer, respectively.
- Sufficient fruit/vegetable consumption and sufficient physical activity were the first and second most powerful protective factors against breast cancer, respectively.
- Ranking and prioritizing risk factors are essential for prevention programs.
- Both the strength of association and the prevalence of risk factors are important for ranking.
